# Association of Life's Simple 7 with mild cognitive impairment in community-dwelling older adults in China: a cross-sectional study

**DOI:** 10.3389/fnagi.2023.1203920

**Published:** 2023-05-24

**Authors:** Mengshu Yang, Yilan Liu, Xiuzhen Hu, Dianxu Ren, Qing Yang, Jing Mao, Jing Chen

**Affiliations:** ^1^School of Nursing, Tongji Medical College, Huazhong University of Science and Technology, Wuhan, Hubei, China; ^2^Department of Nursing, Union Hospital, Tongji Medical College, Huazhong University of Science and Technology, Wuhan, Hubei, China; ^3^Xinmin Community Health Center, Wuhan, Hubei, China; ^4^School of Nursing, University of Pittsburgh, Pittsburgh, PA, United States; ^5^Department of Nursing, Tongji Hospital, Tongji Medical College, Huazhong University of Science and Technology, Wuhan, Hubei, China

**Keywords:** Life's Simple 7, cardiovascular health, mild cognitive impairment, dementia, cardiovascular disease, risk factors

## Abstract

**Background:**

Life's Simple 7 (LS7), a metric composed of seven intervenable cardiovascular risk factors, is initiated by the American Heart Association to improve cardiovascular health. The components of LS7 have been reported as risk factors for dementia. However, few studies investigated the association between LS7 metric and mild cognitive impairment (MCI).

**Methods:**

The study was carried out in a primary care facility between 8 June and 10 July 2022. A total of 297 community-dwelling residents aged 65 years or older were recruited. Sociodemographic, comorbidity, and lifestyle characteristics were collected through the questionnaires, and biological parameters were obtained from blood sample examinations. Logistic regression was used to analyze the association between LS7 scores (overall, behavioral, and biological) and individual components with MCI, adjusting sex, age, education, and cardiovascular disease (CVD).

**Results:**

In comparison with the cognitively intact group (*n* = 195), the MCI group (*n* = 102) had a lower education level and a higher proportion of hypertension. Multivariate logistic regression analysis, adjusting sex, age, education, and CVD demonstrated a significant association between MCI and overall LS7 score [odd ratio = 0.805, 95% confidence interval (0.690, 0.939)] and biological score [odd ratio = 0.762, 95% confidence interval (0.602, 0.965)].

**Conclusion:**

Life's Simple 7 was associated with MCI in community-dwelling older adults, indicating that LS7 could be used as guidance in the prevention of dementia in the community.

## Introduction

With global aging, dementia cases are projected to reach 78 million by 2030, costing US$2.8 trillion annually (Zhang et al., 2021). Although the pathogenesis of dementia remains unclear, it is widely recognized as a multifactorial disease with various risk factors, including obesity, smoking, physical inactivity, poor diet, depression, hypertension, diabetes, hyperlipidemia, and coronary heart disease (Deckers et al., 2015). Some of these risk factors may be interrelated and unchangeable, and current evidence on the combined effect of multiple risk factors is limited, primarily focusing on individual risk factors (Cooper et al., 2015; Xu et al., 2015) or lifestyle factors (Flicker, 2010; Gelber et al., 2012; Sabia et al., 2017; Samieri et al., 2018). Therefore, identifying and addressing the key modifiable risk factors are essential for effective dementia management.

The Lancet Commission and the World Dementia Council recommended targeting cardiovascular risk factors in their guidelines for preventing dementia (Winblad et al., 2016; Livingston et al., 2020). Life's Simple 7 (LS7) metric was first proposed by the American Heart Association (AHA) to define and monitor the prevalence of ideal cardiovascular health (CVH) and reduce the morbidity and mortality from cardiovascular disease (CVD) in the US population. LS7 metric focuses on modifiable cardiovascular risk factors, including four behavioral factors [smoking, diet, physical activity, and body mass index (BMI)] and three biological factors (untreated blood pressure, total cholesterol, and fasting plasma glucose), and the status of these factors are classified into levels of poor, intermediate, and ideal (Lloyd-Jones et al., 2010). In a dose-response meta-analysis by Aneni et al., each increase in the number of ideal CVH components was associated with a pooled hazard ratio for CVD mortality of 0.81 [95% confidence interval (CI), 0.75–0.87; Aneni et al., 2017]. In a meta-analysis by Fang et al., achieving the greatest number of ideal CVH components was associated with a lower risk of CVD (risk ratio = 0.20; 95% CI, 0.11–0.37) and cardiovascular mortality (risk ratio = 0.25; 95% CI, 0.10–0.63; Fang et al., 2016), suggesting LS7 a useful tool for cardiovascular risk assessment. There was a potential mechanism that cardiovascular risk factors were believed to have deleterious effects on the structure and function of cerebral blood vessels, leading to a decrease in cerebral perfusion and promoting disturbances in amyloid clearance, resulting in neurovascular dysfunction and sub-optimal brain health (Gorelick et al., 2017). Moreover, cardiovascular risk factors in LS7 emerge in the aforementioned risk factors for dementia, implicating that LS7 may be put forward as a potential tool for the prevention of dementia.

In 2017, LS7 was identified as the practical criteria for defining brain health, which encompasses cognitive processes such as learning, judgment, communication, and memory (Gorelick et al., 2017). Recently, a statement based on LS7 was issued by AHA, providing an up-to-date summary for primary care physicians to evaluate cardiovascular risk factors, preserve brain health, and prevent cognitive impairment (Lazar et al., 2021). In the previous studies related to dementia, Janice L. Atkins et al. demonstrated that individuals with optimal LS7 profiles had a 33% reduction in risk of incident hospital-diagnosed dementia using population-representative medical records of the UK, where incident CVD events occurred (Atkins et al., 2019). As for preventing cardiovascular morbidity, Langa et al. reported a significant decline in the prevalence of dementia among the US population aged 65 years or older (8.8% in 2012 vs. 11.6% in 2000) in the presence of self-reported heart disease (Langa et al., 2017). As mentioned above, the relationship between CVH and dementia is necessarily linked to the development of CVD. Although most cases of cognitive decline have similar pathogenesis being mixed with contributions by neurodegenerative disease, comorbidities, and CVD (Schneider et al., 2007; Langa and Levine, 2014; Arvanitakis et al., 2019), whether CVH can directly influence cognitive status in the absence of CVD is unclear.

Mild cognitive impairment (MCI) is a clinical diagnosis of a syndrome on the continuum of cognitive decline between normal cognition and dementia (Petersen, 2011; Langa and Levine, 2014). In the symptomatic predementia stage, a consensus has been established that primary intervention in this population can thwart or delay the progression of cognitive deterioration to dementia and decrease the incidence or prevalence of dementia (Jia et al., 2020). However, few studies using LS7 identified the association between the LS7 metric and MCI. Meanwhile, fewer studies have been conducted in Asian countries such as China (Gildner et al., 2018), where the prevalence and growth rate of dementia are the highest, accounting for nearly 25% of all dementia cases worldwide (GBD 2016 Neurology Collaborators, 2019; Zhou et al., 2019). This study aims to investigate whether the LS7 metric, as the combination of changeable risk factors, is associated with the incidence of MCI without CVD in community elderlies in China, providing further evidence for the assessment and management of cognitive risk.

## Methods

### Study design

The study is a cross-sectional, population-based survey carried out in the primary care setting from 8 June to 10 July 2022.

### Study participants

In this study, the community where 2,618 adults live has seven subdivisions. A total of 318 participants in Wuhan, Hubei, China were recruited through convenience sampling.

#### Inclusion criteria

Participants were included if they (1) were aged 65 years or older; (2) accomplished the health check-ups; and (3) signed informed consent.

#### Exclusion criteria

**Figure 1:**
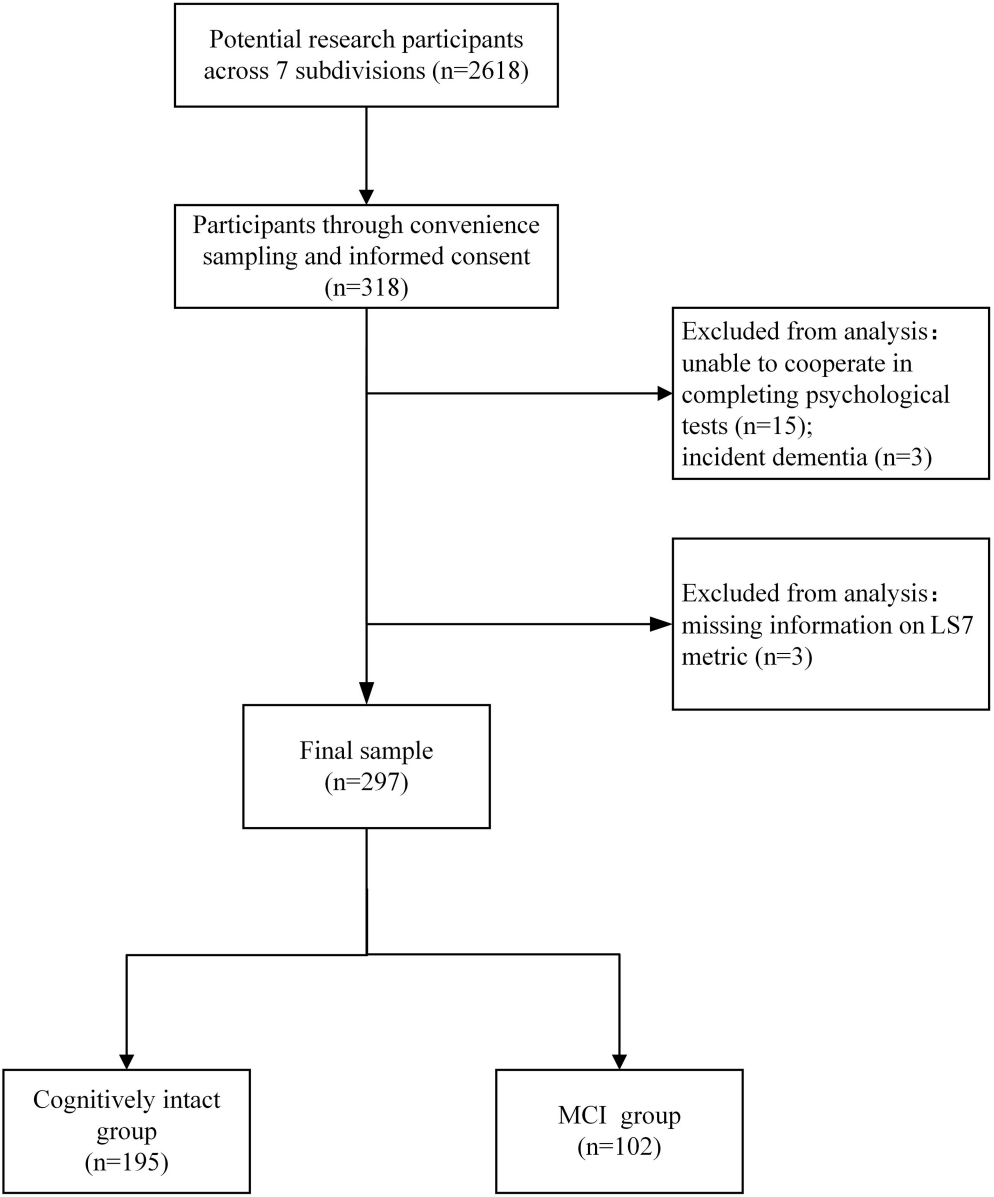
Flow chart of participants.

Participants were excluded if they (1) had an acute illness or undergone surgery in recent 3 months; (2) were unable to cooperate in completing psychological tests due to visual impairment, hearing impairment, or other impairment; (3) were taking medications that might affect cognition or taking anti-psychotic medications (e.g., antidepressants for severe depression); and (4) had a self-reported or recorded diagnosis of neurological disorders (e.g., parkinsonism or incident dementia; see Figure 1).

### Data collection

Sociodemographic characteristics including sex, age, education level, and marital status were collected through the questionnaire. Former alcohol drinkers were defined as participants who quit drinking within <12 months, while those who never drank or quit drinking for more than 12 months were considered non-alcohol drinkers. Similarly, former smokers were defined as participants who quit smoking within <12 months, and those who never smoked or quit smoking for more than 12 months were considered non-smokers. Information on hypertension, diabetes, hyperlipidemia, and CVD was self-reported by participants through the questionnaire and then can be determined by the research team in their medical records. LS7 metric was calculated using de-identified data for all the participants.

### Diagnostic criteria

Mild cognitive impairment scored 0.5 on the global Clinical Dementia Rating scale and was diagnosed according to the following criteria by a physician: (1) the presence of spontaneous cognitive complaints; (2) suggested objective impairment in cognitive domains of memory, executive function, attention, and language by cognitive tests such as Montreal Cognitive Assessment; (3) preserved activities of daily living on the disability scale confirmed by clinician's interviews; and (4) no dementia according to the Diagnostic and Statistical Manual of Mental Disorders (Petersen, 2011). The Chinese version of the Montreal Cognitive Assessment scale (version 7.1) was used in this study. To correct for literacy, participants with ≤12 years of education were added 1 point to their overall scores. The MCI group was scored with <26 points.

Dementia was determined by scoring more than 0.5 on the global Clinical Dementia Rating scale and meeting the Diagnostic and Statistical Manual of Mental Disorders (fifth edition), which required those as follows: (1) memory impairment and impairment in at least one of the other domains such as aphasia, apraxia, agnosia, or executive functioning; (2) impairment and decline in social or occupational function; and (3) cognitive deficits that do not occur exclusively during the course of a delirium episode.

Participants without MCI and dementia were classified as cognitively intact.

### Measurements of LS7 metric

Blood pressure was defined as the average of two consecutive blood pressure readings in the right arm in the seating position. Total cholesterol and fasting plasma glucose were determined using a peripheral blood sample after a minimum of 5 h of fasting. BMI was calculated as weight divided by the square of height (kg/m^2^). Diet, smoking status (current, former, and never), and physical activity (time of moderate and vigorous activity per week) were self-reported. According to the AHA definition and criteria (Lloyd-Jones et al., 2010), each individual component in LS7 was given a score of poor (coded as 0), intermediate (coded as 1), or ideal (coded as 2). In addition, the scoring criteria for diet was modified to the intake of fruit, vegetables and fishes since they may be more available to collect in health check-ups (Samieri et al., 2018; see Supplementary Table 1). Behavioral score, defined as the summarization of scores of diet, smoking, physical activity, and BMI ranges from 0 (worst) to 8 (best). The biological score, defined as the summarization of the scores of blood pressure, total cholesterol, and fasting plasma glucose ranges from 0 (worst) to 6 (best). The overall score of LS7, which ranged from 0 to 14, was divided into three categories: poor [< mean – standard deviation (SD)], intermediate (≥ mean – SD and < SD + mean), and optimal (≥ mean + SD; Sabia et al., 2019).

### Covariates

Covariates included in the analysis were sex, age, education, and related clinical factors. Education level was categorized as “high” (college degree and above) or “low” (high school degree and below).

### Statistical analysis

Participants with missing data were further excluded from data analysis. The Kolmogorov–Smirnov test was used to determine the distribution of continuous variables, demonstrating that age was normally distributed (*P* = 0.579). In univariate analysis, the *t*-test and chi-square test were used for the comparison of continuous variables and categorical variables, respectively. In multivariate analysis, the association of MCI for LS7 metric and individual components was assessed using logistic regression analysis. Interaction analysis was used to evaluate the potential interaction among components by adding a product term. A sensitivity analysis was performed by adjusting for CVD, which was defined as self-reported (verified in medical records) stroke and coronary heart disease and heart failure (Malik et al., 2021; Tin et al., 2022). Meanwhile, collinearity analysis was used to examine the accuracy of the regression model based on LS7. Results of the logistic regression models were reported as odd ratio and 95% CI. All the statistical analyses were performed with SPSS v.26 (IBM, NY, USA).

This study followed the strengthening of the reporting of observational studies in epidemiology (STROBE) guidelines (Vandenbroucke et al., 2007), and the study protocol was approved by the Ethics Committee of Tongji Medical College, Huazhong University of Science and Technology (s906).

## Results

### Demographic characteristics of participants

A total of 297 participants (70.9 ± 4.8 years) were included in this study, with 195 in the cognitively intact group and 102 in the MCI group. The MCI group had a lower education level (*p* < 0.01) and a higher proportion of hypertension (*p* < 0.05) than the cognitively intact group. There were no other significant differences between the two groups (Table 1).

**Table 1:** Demographic characteristics of participants.

**Variables**	**Cognitively normal group (*n* = 195)**	**MCI group (*n* = 102)**	***t*** **/χ^2^-value**	***P***
**Sex**, ***n*** **(%)**			2.680	0.102
Female	103 (52.8)	64 (62.7)		
Age, years (mean ± SD)	70.6 (4.6)	71.4 (5.2)	−1.330	0.185
**Education level**, ***n*** **(%)**			19.820	**0.000**
Low (high school degree and below)	138 (70.8)	95 (93.1)		
High (college degree and above)	57 (29.2)	7 (6.9)		
Married, *n* (%)	171 (87.7)	94 (92.2)	1.389	0.239
**Alcohol consumption**, ***n*** **(%)**			0.098	0.952
Current	12 (6.2)	6 (5.9)		
Former	7 (3.6)	3 (2.9)		
Never	176 (90.3)	93 (91.2)		
**Smoking**, ***n*** **(%)**			0.149	0.699
Current	24 (12.3)	11 (10.8)		
Never	171 (87.7)	91 (89.2)		
Hypertension, *n* (%)	83 (42.6)	56 (54.9)	4.094	**0.043**
Diabetes, *n* (%)	27 (13.8)	22 (21.6)	2.899	0.089
Hyperlipidemia, *n* (%)	18 (9.2)	9 (8.8)	0.013	0.908
CVD, *n* (%)	16 (8.2)	9 (8.8)	0.033	0.855

SD, standard deviation; CVD, cardiovascular disease (stroke, coronary heart disease, and heart failure).

Values in bold mean statistically significant (*p* < 0.05).

### Association between MCI and LS7

In the categorical analysis, the optimal CVH status was associated with a 66.0% lower risk of MCI in comparison with the poor status, while no statistically significant association was found in the intermediate. When the scores (continuous) were used in the analysis, the risk for MCI was reduced by 19.4% per 1-point increment in the overall LS7 score and was reduced by 23.7% per 1-point increment in the biological score after adjustment for sex, age, and education (Table 2).

**Table 2:** Logistic regression modeling the association between MCI and LS7.

	**OR**	**95% CI of OR**
**LS7, per 1-point increment**		
Overall LS7 score	**0.806**	**(0.691, 0.940)**
Behavioral score	0.833	(0.680, 1.021)
Biological score	**0.763**	**(0.603, 0.966)**
**LS7, categories**		
Poor, 0–9	Reference	
Intermediate, 10–12	0.660	(0.351, 1.242)
Optimal, 13–14	**0.340**	**(0.134, 0.860)**

MCI, mild cognitive impairment; LS7, Life's Simple 7; OR, odd ratio; CI, confidence interval.

Models were adjusted for sex, age, and education.

Values in bold mean statistically significant (*p* < 0.05).

### Association between MCI and each individual component

All the participants reached the ideal level for diet according to their self-reported dietary status, and there were no former smokers among the participants. In the adjusted logistic regression models, achieving an ideal level of fasting plasma glucose reduced the risk of MCI by 56.2%, but no other components showed a significant association with MCI (Table 3). Interactions between levels of individual components (categorical) were tested, but no significant interactions are found in Supplementary Table 2.

**Table 3:** Logistic regression modeling the association between MCI and each individual component (the final independent variable entered in each model).

**Components**	**Adjusted OR**	**95% CI of OR**
**Smoking**		
Poor	Reference	
Ideal	1.042	(0.439, 2.472)
**Physical activity**		
Poor	Reference	
Intermediate	0.994	(0.391, 2.525)
Ideal	0.613	(0.343, 1.096)
**Body mass index**		
Poor	Reference	
Intermediate	0.448	(0.112, 1.794)
Ideal	0.363	(0.094, 1.406)
**Total cholesterol**		
Poor	Reference	
Intermediate	1.393	(0.547, 3.545)
Ideal	1.896	(0.784, 4.517)
**Blood pressure**		
Poor	Reference	
Intermediate	1.127	(0.616, 2.061)
Ideal	0.642	(0.335, 1.231)
**Fasting plasma glucose**		
Poor	Reference	
Intermediate	0.708	(0.368, 1.363)
Ideal	**0.438**	**(0.206, 0.932)**

OR, odd ratio; CI, confidence interval.

Models were adjusted for sex, age, and education.

Values in bold mean statistically significant (*p* < 0.05).

### Sensitivity analysis

In the sensitivity analysis (Table 4), adjusting for CVD as another covariate showed that the overall score, biological score, and ideal fasting plasma glucose were significantly associated with MCI (per 1-point increment of overall LS7 score reduced by 19.5% lower risk, biological score with 23.8% risk for MCI, fasting plasma glucose with 56.4% risk of MCI). The beta coefficients for the overall score, behavioral score, and biological score are shown in Supplementary Table 3. The results were similar to the prior logistic regression models, demonstrating the robustness and accuracy of this study. We also found no collinearity between CVD and LS7 metrics with all VIFs < 5 (see Supplementary Table 4).

**Table 4:** Results of sensitivity analysis adding CVD as a covariate.

	**Adjusted OR**	**95% CI of OR**
**LS7, per1-point increment**		
Overall score	**0.805**	**(0.690, 0.939)**
Behavioral score	0.832	(0.679, 1.020)
Biological score	**0.762**	**(0.602, 0.965)**
**LS7, categories**		
Poor, 0–9	Reference	
Intermediate, 10–12	0.661	(0.351, 1.243)
Optimal, 13–14	**0.340**	**(0.135, 0.861)**
**Smoking**		
Poor	Reference	
Ideal	1.054	(0.442, 2.510)
**Physical activity**		
Poor	Reference	
Intermediate	1.014	(0.397, 2.586)
Ideal	0.611	(0.342, 1.093)
**Body mass index**		
Poor	Reference	
Intermediate	0.438	(0.109, 1.755)
Ideal	0.353	(0.091, 1.372)
**Total cholesterol**		
Poor	Reference	
Intermediate	1.387	(0.545, 3.528)
Ideal	1.931	(0.802, 4.650)
**Blood pressure**		
Poor	Reference	
Intermediate	1.075	(0.591, 1.954)
Ideal	0.616	(0.323, 1.175)
**Fasting plasma glucose**		
Poor	Reference	
Intermediate	0.701	(0.363, 1.353)
Ideal	**0.436**	**(0.205, 0.927)**

LS7, Life's Simple 7; OR, odd ratio; CI, confidence interval.

Models were adjusted for sex, age, education, and cardiovascular disease.

Values in bold mean statistically significant (*p* < 0.05).

## Discussion

Results from this cross-sectional study demonstrated that the optimal category of LS7 reduced the odds of MCI by 66.0% compared with the poor category. The risk for MCI was reduced by 19.4% per 1-point increment in the overall LS7 score and 23.7% per 1-point increment in the biological score. Among the components of LS7, the ideal level of fasting plasma glucose reduced the risk of MCI by 56.2%.

Since dementia is a multifactorial disease with various risk factors, its disease-modifying medications lack effectiveness. For example, aducanumab is only suitable for early dementia patients, but it is still unable to intervene when the disease progresses to the middle and late stages (Sevigny et al., 2016). Identifying and addressing the key modifiable risk factors are of great importance in the prevention of it. This study demonstrated that a higher LS7 score was associated with a reduced risk of MCI, a preclinical stage of dementia, suggesting that the LS7 metric can provide valuable guidance in the risk management of dementia. In consistence, a cohort study in France found that community dwellers aged at least 65 years with a higher LS7 score were linked to a lower risk of attenuated cognitive decline (Samieri et al., 2018). Retrolongitudinal studies demonstrated that higher LS7 overall and biological scores in midlife (aged 45–65 years) were associated with a lower incidence of cognitive impairment at an older age (Thacker et al., 2014; Gonzalez et al., 2018; Malik et al., 2021).

In the categorical analysis of our study, the optimal category (13–14) of LS7 demonstrated a substantial reduction in the risk of MCI, but no significant change in the risk of MCI was found in the intermediate category (10–12) in community-dwelling old adults. A study in adults aged 65 years or older in Northern Manhattan showed that both intermediate (6–9) and optimal category (10–14) of LS7 were associated with reduced incidence of dementia (Guo et al., 2021). We guess that the difference in the outcome variable (MCI vs. dementia) may account for the discrepancy. In the Framingham Heart Study Offspring cohort, higher recent CVH scores were associated with less cognitive impairment and a lower 10-year risk of incident stroke, but there was no association with incident all-cause dementia or Alzheimer's disease. Higher remote CVH scores were all associated with a lower 10-year risk of incident stroke, dementia, and less cognitive impairment (Pase et al., 2016). Accordingly, our results remain robust when CVD is adjusted. These findings suggested that the LS7 components might contribute to the development of both CVD and cognitive decline simultaneously, and CVD is not a necessary mediating factor in the development of dementia but rather a concurrent outcome.

Although a significant association was observed for the LS7 overall or biological score with a decreased MCI risk, no significant association was found for the behavioral score. Several reasons may be involved. First, all the participants self-reported an ideal-level diet, and no participants were former smokers in our study. Due to a lack of diversity in the social demographic characteristics within the study population, it is difficult to identify the impact of behavioral dimensions. Second, the recall or comprehension bias of self-reported lifestyle may affect the results (Gardener et al., 2016). Third, the effects of behavior may already be present in the biological status. However, further research studies on a larger population are still needed to clarify the underlying reasons.

Fasting plasma glucose, which has been shown to influence the onset and progression of the many underlying pathologies associated with dementia (Biessels et al., 2006), was the only component that showed a significant association with MCI incidence, confirming that diabetes or poor fasting plasma glucose control in older age was a major risk factor accelerating cognitive decline and dementia (Yaffe et al., 2012; Biessels et al., 2014). Mechanism studies have explained the effect of the plasma glucose level on brain function. Prolonged exposure to hyperglycemia would lead to abnormal cerebral capillaries that impair brain perfusion (Gispen and Biessels, 2000). Insulin, which would elevate insulin resistance, is actively carried across the blood–brain barrier (Banks, 2004) and activated via cerebral insulin receptors (Bondy and Cheng, 2004), affecting the energy homeostasis in the brain and interfering the learning and memory (Zhao and Alkon, 2001). Moreover, alterations of insulin and glucose homeostasis in the brain may influence amyloid metabolism by stimulating its secretion and blocking its breakdown (Craft and Watson, 2004).

It is uncertain why other individual components reported in studies of other populations were not significantly associated with MCI in this sample. In particular, the contentious “obesity paradox” pointed to the protective effects of high adiposity in later life, and high adiposity may also be harmful in the subsequent period, especially in the presence of other co-existing cardiovascular risk factors (Anstey et al., 2011; Qizilbash et al., 2015). Thus, the link between obesity and dementia risk still needs a thorough evaluation. For the unexpected results of individual components, we speculate that it is due to the participant characteristics reducing our capability to detect the impact of these components on cognitive function. For example, a large proportion of participants in our study reported never having smoked (88.2%) in their lifetime, and it was possible that smoking did not accurately capture the risk of MCI, resulting in discrepancy from a cross-sectional study in China (Jia et al., 2020).

## Limitations

The cross-sectional design could not induce cause–effect relationship, and the small sample size restricted its capacity to be generalized. Selection bias may exist since the small sample size and older adults with cognitive impairment, especially those with psychological symptoms, may be less likely to participate in the study. A cohort study with a larger sample and longer follow-up is needed to confirm the reliability of the LS7 metric and its causality with MCI.

## Implications

As risk factor management for dementia is typically handled by general practitioner providers in most countries, and LS7 consists of common primary care information, it would be a practical strategy to focus on educating and inspiring older adults to adhere to LS7 recommendations in order to maintain their cognitive health.

## Conclusion

In conclusion, our study discovered a strong association between a higher LS7 score and optimal CVH status with a substantially attenuated risk of MCI. LS7 was able to reveal valuable information that was not apparent in the individual components, even in a small sample, suggesting that it captures several key risk factors for cognitive decline. LS7 could be an effective and convenient tool for medical staff to monitor and manage geriatric cognitive health in both research and clinical practice. Future research investigating whether interventions targeting LS7 components can prevent or reverse dementia is suggested.

## Data availability statement

The original contributions presented in the study are included in the article/Supplementary material, further inquiries can be directed to the corresponding authors.

## Ethics statement

The study protocol was approved by Ethics Committee of Tongji Medical College, Huazhong University of Science and Technology (s906). The patients/participants provided their written informed consent to participate in this study.

## Author contributions

JM and MY contributed to the conception and design of the study. YL, QY, and JC provided clinical knowledge support and constructive suggestions for the study. MY, XH, and JC collected and assessed the data. MY and XH analyzed and interpreted the data. DR provided critical guidance on data analysis. MY and YL drafted the manuscript. All authors contributed to the manuscript revision and approved the submitted version.
