# Supplementary material for: Association of Life's Simple 7 with mild cognitive impairment in community-dwelling older adults in China: a cross-sectional study
Source: Front Aging Neurosci. 2023 May 24;15:1203920. doi: 10.3389/fnagi.2023.1203920 (PMC10244635; doi:10.3389/fnagi.2023.1203920)
Supplement: Supplementary file 1 [file Table_1.DOCX]

Supplementary Material

## Supplementary tables

**Supplementary Table 1. Definition of Life’s Simple 7**

|  | Poor | Intermediate | Ideal |
| --- | --- | --- | --- |
| Diet | ≥1portion per d of each of fresh fruit, raw vegetables, cooked fruit/vegetables and ≥2 portions per wk of fish | ≥1 portion per d of fresh fruit, raw vegetables, cooked fruit/vegetables or ≥2 portions per wk of fish | <1 portion per d of fresh fruit, raw vegetables, cooked fruit/vegetables, and <2 portions per wk of fish |
| Physical activity | No physical activity | 1-149 minutes/week of moderate activity, or 1-74 minutes/week of  vigorous activity, or 1-149 minutes/week of moderate and vigorous activity | ≥150 minutes/week of moderate activity, or ≥75 minutes/week of vigorous activity, or ≥150 minutes/week of moderate and vigorous activity |
| Smoking | Current smoking | Quitting smoking for less than 12 months | Never smoking or quit smoking for more than 12 months |
| Body mass index | BMI ≥30kg/m^2^ | BMI 25-29.9kg/m^2^ | BMI <25kg/m^2^ |
| Total cholesterol | Total cholesterol ≥240mg/dL | Total cholesterol 200-239mg/dL | Total cholesterol <200mg/dL |
| Blood pressure | Systolic blood pressure ≥140 mmHg and diastolic blood pressure≥90 mmHg | Systolic blood pressure  120-139 mmHg, or  diastolic blood pressure  80-89 mmHg, or treated to goal | Systolic blood pressure  <120 mmHg and diastolic  blood pressure <80mmHg |
| Fasting plasma glucose | FPG≥126mg/dL | FPG 100-125 mg/dL | FPG <100 mg/dL |

Abbreviations: BMI, body mass index; FPG, fasting plasma glucose.

**Supplementary Table 2. Interactions between CVH components (categorical).**

| Components | *P* _interaction_ | | | | | |
| --- | --- | --- | --- | --- | --- | --- |
|  | Smoking | Physical activity | Body  mass index | Total cholesterol | Blood pressure | Fasting plasma glucose |
| Smoking | --- | 0.634 | 0.680 | 0.257 | 0.226 | 0.765 |
| Physical activity | 0.634 | --- | 0.183 | 0.553 | 0.791 | 0.824 |
| Body mass index | 0.680 | 0.183 | --- | 0.945 | 0.784 | 0.882 |
| Total cholesterol | 0.257 | 0.553 | 0.945 | --- | 0.154 | 0.700 |
| Blood pressure | 0.226 | 0.791 | 0.784 | 0.154 | --- | 0.906 |
| Fasting plasma glucose | 0.765 | 0.824 | 0.882 | 0.700 | 0.906 | --- |

**Supplementary Table 3. The value of coefficients with and without CVD incorporated into the regression models on L7 metrics.**

|  | Coefficient^a^ | Coefficient^b^ |
| --- | --- | --- |
| LS7, per 1-point increment |  |  |
| Overall LS7 score | -0.215 | -0.217 |
| Behavioral score | -0.183 | -0.184 |
| Biological score | -0.270 | -0.272 |
| LS7, categories |  |  |
| Poor, 0-9 | Reference | Reference |
| Intermediate, 10-12 | -0.415 | -0.414 |
| Optimal, 13-14 | -1.079 | -1.078 |

Abbreviation: LS7, Life’s Simple 7.

Corner label a means models were adjusted for sex, age, and education.

Corner label b means models were adjusted for sex, age, education, and cardiovascular disease.

**Supplementary Table 4. The results of collinearity diagnosis.**

|  | β | t value | *P* | VIF |
| --- | --- | --- | --- | --- |
| Overall score | -0.160 | -2.835 | **0.005** | 1.035 |
| Sex | -0.078 | -1.331 | 0.184 | 1.120 |
| Age | 0.120 | 2.114 | **0.035** | 1.055 |
| Education level | -0.255 | -4.411 | **0.000** | 1.087 |
| CVD | -0.029 | -0.514 | 0.607 | 1.027 |
| Behavioral score | -0.103 | -1.809 | 0.071 | 1.037 |
| Sex | -0.069 | -1.160 | 0.247 | 1.123 |
| Age | 0.128 | 2.220 | 0.027 | 1.058 |
| Education level | -0.255 | -4.378 | **0.000** | 1.090 |
| CVD | -0.025 | -0.438 | 0.662 | 1.026 |
| Biological score | -0.129 | -0.297 | **0.022** | 1.015 |
| Sex | -0.058 | -0.992 | 0.322 | 1.091 |
| Age | 0.112 | 1.953 | **0.052** | 1.061 |
| Education level | -0.264 | -4.557 | **0.000** | 1.083 |
| CVD | -0.027 | -0.478 | 0.633 | 1.027 |

Abbreviations: CVD, cardiovascular disease; β, parameter estimates; VIF, variance inflation factor.

Values in bold mean statistically significant (*p* < 0.05).
